# *Escherichia coli *MW005: lambda Red-mediated recombineering and copy-number induction of *oriV*-equipped constructs in a single host

**DOI:** 10.1186/1472-6750-10-27

**Published:** 2010-03-29

**Authors:** Marcel Westenberg, Sophie Bamps, Helen Soedling, Ian A Hope, Colin T Dolphin

**Affiliations:** 1 Pharmaceutical Science Division, King's College London, 150 Stamford Street, London, SE1 9NH, UK; 2 Institute of Integrative and Comparative Biology, Faculty of Biological Sciences, The University of Leeds, Leeds, LS2 9JT, UK

## Abstract

**Background:**

*Escherichia coli *strain EL350 contains chromosomally integrated phage lambda Red recombinase genes enabling this strain to be used for modifying the sequence of resident clones *via *recombineering. BAC and fosmid clones are highly suitable for modification by recombineering but, because they are present at low (1-2) copies per cell, the DNA is difficult to isolate in high yield and purity. To overcome this limitation vectors, e.g. pCC1FOS, have been constructed that contain the additional replication origin, *oriV*, which permits copy-number to be induced transiently when propagated in a suitable host strain, e.g. EPI300, that supplies the cognate *trans*-replication protein TrfA. Previously, we used EL350 and EPI300 sequentially to recombineer *oriV*-equipped fosmid genomic clones and, subsequently, to induce copy-number of the resulting recombinant clone. To eliminate these intervening DNA isolation and transformation steps we retrofitted EL350 with a *P*_BAD_-driven *trfA *gene generating strain MW005 that supports, independently, both recombineering and copy-number induction.

**Results:**

The *P*_BAD_-driven copy of *cre *in EL350 was replaced seamlessly with a copy of *trfA*, PCR-amplified from EPI300 chromosomal DNA, to generate MW005. This new strain has been used to both generate, via recombineering, a number of reporter gene fusions directly from pCC1FOS-based *Caenorhabditis elegans *genomic clones and to transiently induce copy-number of fosmid and BAC clones prior to DNA preparation.

**Conclusions:**

By retrofitting EL350, an established 'recombineering' *E. coli *strain, with a tightly regulated copy of *trfA *we have produced a new strain, MW005, which combines recombineering capacity with the useful ability to transiently induce copy-number of *oriV*-equipped clones. By coupling these two steps in a single strain, use of MW005 will enable the more rapid recombineering-mediated production of recombinant clones in the yield and quality necessary for many downstream purposes.

## Background

Recombineering (**recombin**ogenic engin**eering**) is a relatively recently described technique of homologous recombination (HR)-based genetic engineering performed within an *E*. *coli *host and mediated by transient expression of phage-encoded recombinases [reviewed in ref [1]]. Both lambda Red [2] and the Rec E/T [3] recombinase systems have been developed into recombineering tools for transient, controlled expression of the respective recombinase activities. Whilst recombineering can be used to modify the host chromosome the technique is more commonly applied to episomal replicons including low-copy-number BACs or fosmids and intermediate and multicopy plasmids. Modifications are targeted and precise and can range from single base-pair deletions or insertions to the addition or deletion of sequences in the kilobase-pair range. Bacteria containing the target are transformed with linear, double- or single-stranded, donor DNA molecules bearing the desired sequence changes. These donor molecules are generated, as PCR-amplicons, restriction fragments or oligonucleotides, that contain, at each end, appropriate regions of homology to the circular target. In contrast to RecA-mediated genetic engineering only short (~50 bp) homologies are required to mediate efficient HR between donor and target molecules. These "homology arms" can be built easily into a donor by PCR. Recombineering is particularly useful when manipulating larger target molecules, such as BACs or fosmids, which, because of their size, will likely lack the required range of unique and appropriately positioned restriction enzyme sites necessary to achieve subtle modifications. Recombineering is gaining wider acceptance as a genetic engineering tool and may be viewed as a viable alternative to more traditional restriction enzyme/ligase-based approaches particularly when modifying large target molecules.

The nematode *C. elegans *is a genetically tractable model animal that, since its initial introduction [4], has become the experimental system of choice for many laboratories. Determining the expression pattern for a gene-of-interest (*goi*) by analysing β-galactosidase or fluorescent protein reporter expression in *C*. *elegans *transformed with a *goi*::*reporter *fusion gene is a commonly performed procedure. In a so-called transcriptional reporter gene fusion the expression of the reporter protein alone is driven by DNA fragments known, or presumed, to contain all or part of the *goi's *promoter. In *C. elegans *this usually comprises 1-4 kb from immediately 5' of the translational start codon. Such constructions will, by definition, exclude any regulatory elements that lie outside the assayed genomic DNA fragment. In contrast, in a translational reporter gene fusion the reporter gene is fused, in-frame, to all, or part, of the *goi's *protein coding region to encode a fusion protein. Such a translational reporter gene fusion would, ideally, contain not only all in the immediate vicinity of the *goi's *protein coding region but also significant stretches of 5' and 3' flanking DNA, and so include more distantly located regulatory elements.

As discussed, recombineering is ideal for engineering large targets and a number of protocols designed to generate translational-style reporter gene fusions directly from *C. elegans *genomic clones have been described [5-8]. Additionally, we [5], and others [7], have combined recombineering with counter-selection to enable the reporter sequence to be inserted seamlessly into the target. Such counter-selection strategies are, however, limited to fosmid or other low-copy-number vector targets because the relative inefficiency of the recombineering method makes it difficult otherwise to select negatively for desired recombinants. The *C. elegans *genome is covered extensively with genomic clones from a library constructed in the copy-number-inducible fosmid vector pCC1FOS (CopyControl, Epicentre, Madison, USA; Moerman D., pers. comm.). In addition to the F factor system, that maintains pCC1FOS-based clones at 1-2 copies per chromosome, pCC1FOS also contains an *oriV *replication origin. When propagated in a suitable host strain, e.g. EPI300 (Epicentre), that carries *trfA *encoding the associated replication protein TrfA, the *oriV *replication origin allows copy-number to be induced to approximately 50-100 copies per host chromosome. If *trfA *transcription is tightly controlled, for example by the L-arabinose-regulated *araC-P*_BAD _system, then *oriV*-equipped fosmid copy-number is only increased "on-command" when high yields of good-quality DNA need to be isolated for downstream events [9].

The counter-selection protocol applied by us [5,10] utilises *E. coli *host strains, e.g. EL250 and EL350 [11] and derivatives there from, that provide recombineering capacity *via *a chromosomally integrated defective lambda prophage. The prophage contains the Red recombinase genes arranged in their natural context and tightly regulated by the temperature-sensitive cI857 repressor [2]. Although recombinase functions can be supplied [for example ref [12]] *via *plasmids the tighter control and coordinated expression of the integrated prophage approach is considered more efficient and controllable. Our counter-selection approach [5] utilizes a dicistronic counter-selection cassette (the RT-cassette), containing the respective positive and negative markers *tet*A(C) and *rps*L driven by the hypo-osmotically up-regulated *omp*F promoter to provide stringent positive and negative selection for single-copy vectors [13]. The RT-cassette, inserted at the target site by an initial recombineering step using positive selection for recombinants, is replaced with the desired sequence in a second round of recombineering using negative selection. Positive selection is provided by *tet*A(C) conferring tetracycline (Tc) resistance (Tc^R^) whereas, when expressed in a *rps*L^- ^host, the wild-type *rps*L^+ ^gene provides the negative marker. Mutations in the chromosomal *rps*L, that encodes the ribosomal protein S12, confers streptomycin [Sm], resistance (Sm^R^). When both mutant and wild-type *rps*L alleles are co-expressed a dominant Sm sensitive (Sm^S^) phenotype results. Because EL250 and EL350 are *rps*L^- ^and thus Sm^R^, the introduction, via the RT-cassette, of a *rps*L^+ ^allele confers Sm^S^. Consequently, non-recombinants following the second recombineering step will remain Sm^S ^and are selected against whereas desired recombinants are Sm^R ^revertants.

Whilst efficient in mediating recombineering, a strain such as EL350 does not permit copy-number induction of resident *oriV*-equipped clones. Thus, our present protocol requires an additional fosmid DNA isolation step and transformation into a TrfA-producing strain to enable transient copy-number induction. In order to combine recombineering and copy-number induction in a single strain we have retrofitted the chromosome of EL350 with a copy of *trfA *under control of the *araC-P*_BAD _regulatory system. The resulting strain, MW005, enables recombineering and copy-number induction to be performed in a single host with significant improvements in speed and productivity. We have used MW005 successfully to create a number of reporter gene fusions in pCC1FOS-based genomic clones. We describe here construction details for MW005 and demonstrate its use in supporting controlled copy-number induction of *oriV*-equipped fosmid and BAC genomic clones. In addition, we also illustrate its use in recombineering in the construction of a pCC1FOS-based translational GFP reporter gene fusion for the *C. elegans *transcription factor gene *ceh-12*.

## Results and Discussion

**Figure 1:**
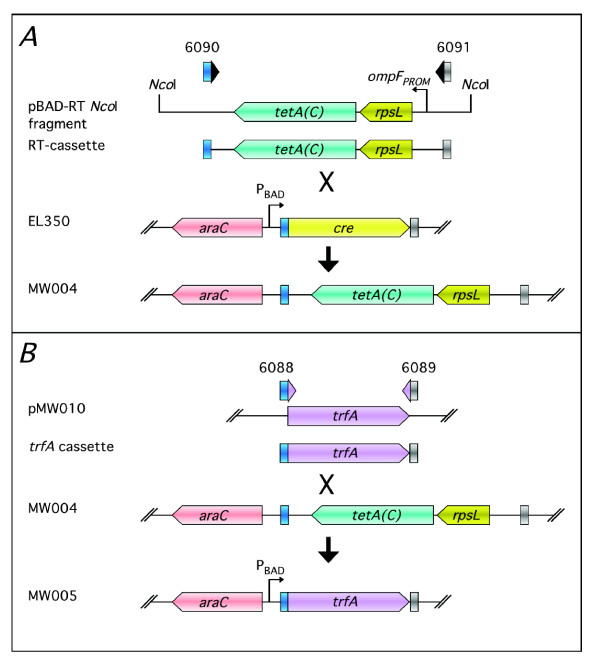
**Recombineering-mediated construction of *E*. *coli *strain MW005**. **Panel A**. An *rps*L-*tet*A(C) counter-selection cassette, PCR-amplified from an *Nco*I fragment of pBAC-RT using ODNs 6090/6091 (Table 1) and flanked with 50-nt homology arms, was used to replace, by recombineering, the chromosomal copy of *cre *in *E*. *coli *strain EL350 to give strain MW004. **Panel B**. A *trfA *replacement cassette, PCR-amplified from pMW010 using ODNs 6088/6089 (Table 1) and flanked with the same 50-nt homology arms as the RT-cassette, was used to replace the RT-cassette in MW004 with the *trfA *gene sequence to give MW005.

The "on-command" *oriV*/TrfA induction system [9] enables the copy-number of large genomic DNA clones, based in vectors such as BACs or fosmids, to be either maintained at 1-2 copies per cell or be increased transiently to 50-100 copies per cell. Low copy-number ensures clone stability while high copy-number provides for increased yields and improved DNA purity at DNA isolation. Controlled copy-number regulation requires the vector be equipped with the *oriV *replication origin and be propagated in a suitable *E*. *coli *host that carries an inducible copy of the *trfA *gene encoding the *trans*-acting replication protein TrfA. Suitable vectors, e.g. pCC1BAC or pCC1FOS, and *trfA*-containing strains, e.g. EPI300, are available commercially (CopyControl, Epicentre). We have described previously [5,10] recombineering protocols designed to generate large translational-style reporter gene fusions directly in *C*. *elegans *genomic clones from a pCC1FOS-based genomic DNA library. However, these protocols require that, following clone engineering in a recombineering strain, final recombinants are moved back into EPI300, the original library host strain, for subsequent copy-number induction and isolation of fosmid DNA. To eliminate this step we utilised our counter-selection recombineering strategy to replace seamlessly the chromosomal *P*_BAD_-driven *cre *in the recombineering strain EL350 [11] with a copy of *trfA *(Fig. 1) to generate strain MW005 (F- *mcrA *Δ(*mrr-hsd*RMS-*mcrBC*) Φ80d*lac*Z M15 *Δlac*X74 *deo*R *rec*A1 *end*A1 *ara*D139 Δ(*ara*, *leu*) 7649 *gal*U *gal*K *rsp*L *nup*G [*λcI*857 (*cro*-*bio*A) < >*ara*C-PBAD*trfA*]). MW005 enables both recombineering and copy-number induction to be performed in the same host. The *trfA *DNA, PCR-amplified from EPI300, encodes the full-length (382 amino acid) TrfA protein and contains the G254D "copy-up" mutation demonstrated to increase significantly copy-number induction [9,14].

**Figure 2:**
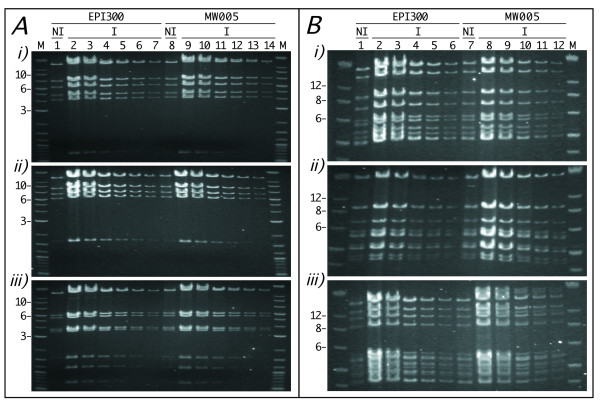
**Copy-number induction in MW005**. **Panel A**. Aliquots (5 μl) of fUL#SB28 (gel i), WRM0636aA04 (gel ii) or WRM067aC01 (gel iii) pCC1FOS-based DNA, isolated from equivalent numbers of cells from either non-induced (NI) or copy-number-induced (I) cultures of EPI300 or MW005 and incubated with either *Bam*HI (gel i) or *Nco*I (gels ii & iii), were electrophoresed through a 0.7% (w/v) agarose gel either undiluted (lanes 1, 8) or after 2- (lanes 2, 9), 4- (lanes 3, 10), 8- (lanes 4, 11), 16- (lanes 5, 12), 32- (lanes 6, 13) or 64-fold (lanes 7, 14) dilution. **Panel B**. Aliquots (5 μl) of A02_CBP0333 (gel i), A10_CBP1191 (gel ii) or H12_CBP0642 (gel iii) pCC1BAC-based DNA, isolated from equivalent numbers of cells from either non-induced (NI) or copy-number-induced (I) cultures of EPI300 or MW005 and incubated with *Nco*I, were electrophoresed through a 0.7% (w/v) agarose gel either undiluted (lanes 1, 7) or after 2- (lanes 2, 8), 4- (lanes 3, 9), 8- (lanes 4, 10), 16- (lanes 5, 11) or 32-fold (lanes 6, 12) dilution. M = DNA ladder (kb).

To investigate whether MW005 would support copy-number induction of *oriV*-equipped clones we compared copy-number induction in EPI300 and MW005 for three pCC1FOS-based clones, the final recombineered *C*. *elegans *gene fusion reporter fUL#SB28 (see below) plus two native genomic clones, and three pCC1BAC-based genomic clones from a library constructed with *Lates calcarifer *(Barramundi) genomic DNA (kind gift of G.H. Yue). DNAs, isolated from equal numbers of cells from EPI300 or MW005 cultures that were either non-induced for copy-number or had received L-arabinose to drive TrfA expression and thus induce copy-number, were restricted and electrophoresed. Copy-numbers of pCC1FOS- and pCC1BAC-based clones were induced in both EPI300 and MW005 by an approximately equal extent (Fig. 2). For each clone, careful visual comparison between the ethidium bromide-stained restriction fragments of DNA isolated from the control culture with those of a 2-fold serial dilution of the equivalent fragments of DNA isolated from the induced culture enabled fold-induction to be estimated for both strains. Such examination indicates that the copy-numbers of all three pCC1FOS-based clones were induced from 50-60-fold in both the commercial EPI300 strain and MW005, e.g. in Fig. 2A, gels i, ii and iii, compare lane 7, containing the restriction digest, diluted 1/64, from induced EPI300, with lane 1, containing the undiluted restriction digest from non-induced EPI300, and lanes 14 and 8 containing, respectively, the equivalent restriction digest dilutions for induced and non-induced MW005. Although, for the three pCC1BAC-based clones, there was some slight variation in copy-number induction between clones and strains, visual examination of Fig. 2B indicates that, for each BAC clone, copy-number was increased approximately 15-20-fold, e.g. compare lane 5, containing the 1/16-diluted restriction digest from induced EPI300, with lane 1, containing the undiluted restriction digest from non-induced EPI300, and lanes 11 and 7 containing the equivalent dilutions for induced and non-induced MW005, respectively.

**Figure 3:**
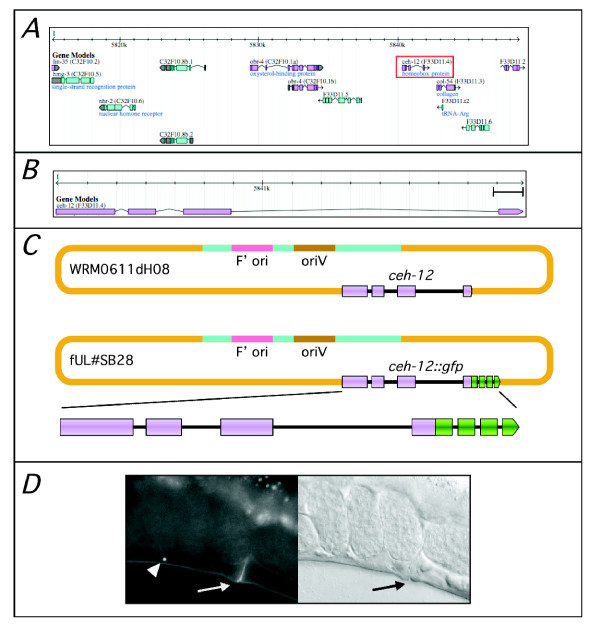
**Recombineering-mediated construction of a fosmid-based *ceh-12*::*gfp *translational reporter gene fusion in MW005**. **Panel A**. A 33.5 kb stretch of *C*. *elegans *chromosome I, from bps 5,815,150 to 5,848,663, equivalent to the insert of genomic clone WRM0611dH08, illustrating the number and orientation of genes predicted within this region (*ceh-12 *boxed in red). **Panel B**. Expanded view of *ceh-12 *illustrating the internal exon- (magenta box) intron (line) organization (scale bar = 100 bp). **Panel C**. Cartoon (not to scale) representing the pCC1FOS-based *C*. *elegans *genomic target clone WRM0611dH08 and the final recombineered construct fUL#SB28. A *gfp *reporter sequence containing four exons (green boxes) was seamlessly inserted, by counter-selection recombineering in MW005, at the 3' end of the *ceh-12 *gene contained within WRM0611dH08 to give fosmid clone fUL#SB28 containing *ceh-12::gfp*. **Panel D**. Expression of *ceh-12*::*gfp *is restricted to VB motorneurons in the ventral nerve cord (arrowhead, left panel fluorescence image). Arrows indicate the vulva on the ventral side of the worm. (Micrographs were captured with Chroma Technology Corp. filter set 41017 on a Leica DMR microscope equipped with DIC optics, a Hamamatsu ORCA ER digital camera and Improvision Openlab software at 400× magnification with a 2 sec exposure). The right panel shows a DIC brightfield image of the same specimen.

We have now used MW005 in a number of experiments in which both recombineering and copy-number induction were performed. One typical procedure involved inserting a *gfp *reporter at the end of the protein coding region of the *C*. *elegans ceh-12 *gene within a pCC1FOS-based genomic DNA clone (Fig. 3). The original clone contained the entire *ceh-12 *locus plus substantial flanking sequences that would likely include more distantly located regulatory elements (Fig. 3A). Following successful *gfp *insertion (Fig. 3C) direct induction of the resulting clone copy-number provided, from a single overnight miniculture, quantities of high-quality DNA sufficient for numerous *C*. *elegans *transformations (data not shown and Fig. 2D). The resulting expression of GFP in VB motorneurons, which innervate ventral body muscles and are involved in forward locomotion, was in accordance with previous investigations of *ceh-12 *gene expression (Fig. 2D) [15].

The coupled pCC1FOS-based genomic clone recombineering and copy-number induction described here have validated the use of MW005 as a convenient host strain in which to perform both recombineering-based genetic engineering and copy-number induction. As there is some evidence for very low levels of "leaky" *cre *expression in EL350 [16] we were concerned initially that if *trfA *expression was similarly leaky in MW005 this may result in pCC1FOS-based clone copy-number being increased above its normal range of 1-2 copies per cell with knock-on effects for clone stability and/or recombineering efficiency. However, restriction enzyme digestion of fosmid and BAC clones isolated from MW005 minicultures (Fig.2 and data not shown) indicated these clones were stably propagated in this strain. In addition to the clone modification described here, MW005 has been used successfully to perform recombineering on more than twenty additional pCC1FOS-based *C*. *elegans *genomic clones and there is no evidence that recombination efficiency differs from the parental strain EL350. Estimates of such efficiency (number of colonies with selection/total viable cell count) × 100 indicate a range of approx. 0.01-0.05% for both EL350 and MW005.

The only experimental proviso on the use of MW005 is that incubation temperatures must not exceed 32°C at any time except during the brief heat-shock needed to de-repress transcription of the Red gene cluster. This is not a significant inconvenience as culture growth rates are slowed only marginally. As we retrofitted strain EL350, MW005 cannot be used for recombineering with a *galK *counter-selection cassette [17] nor can it be used to excise loxP-flanked sequences as the chromosomal copy of *cre *is now replaced with *trfA*. We believe MW005, available from the non-profit plasmid repository Addgene, will prove useful to a number of labs using recombineering to modify *oriV*-equipped clones. For clones lacking an *oriV *this sequence can be easily retrofitted into the clone either by recombineering or by standard genetic engineering techniques or, and perhaps more straightforwardly, via random Tn5 transposon-mediated insertion [18] (EZ-Tn5 system, Epicentre).

## Conclusion

We have described the construction of a new *E. coli *strain, MW005, retrofitted with a *P*_BAD_-driven copy of *trf*A encoding the *trans*-activating replication protein TrfA. Use of MW005 permits researchers to perform both lambda Red-mediated recombineering and subsequent copy-number induction of recombinant DNAs, if these are equipped with *oriV*, in a single host thereby negating the requirement for additional clone isolation and transformation steps. When large number of clones are being modified by recombineering this can translate into significant time saving.

## Methods

### Generation of MW005

#### PCR-amplification and cloning of *trfA*

*E. coli *strain EPI300 (Epicentre, Madison, USA) genomic DNA (gDNA) was isolated using a commercial gDNA isolation kit (DNeasy, Qiagen, Crawley UK) according to the manufacturer's instructions. The gDNA (10 ng) was used as template in a PCR containing oligonucleotides (ODNs) 6075 and 6076 (Table 1) designed to amplify the chromosomal *trfA *gene. The resulting PCR product was cloned in pGEM-T Easy (Promega, Southampton, UK) to give, following confirmation of insert sequence fidelity, pMW010.

**Table 1:** Oligonucleotide sequences

No	Sequence (5'-3')^a^
6075	CTAGCGTTTGCAATGCACCAGGTC
6076	GACGCTTTTTATCGCAACTCTCTAC
6088	ctctactgtttctccatacccgttttttgggctaacaggaggaattaaccATGAATCGGACGTTTGACCGGAAG
6089	atctgtatcaggctgaaaatcttctctcatccgccaaaacagccaagcttCTAGCGTTTGCAATGCACCAGGTC
6090	ctctactgtttctccatacccgttttttgggctaacaggaggaattaaccTCGCTGTCGAGATATGACGGTG
6091	atctgtatcaggctgaaaatcttctctcatccgccaaaacagccaagcttGATGATAAGCTGTCAAACATGAG
6102	CACCGTGCGTGTTGACTATTTTACC
6103	ACGATGTGCGCGTACTGGGGGC
S0316	aaaacaaacgttgcccatcttcaactccaattcaatcaacttcctcttctATGAGTAAAGGAGAAGAACT
S0317	ttgattaagaatttattaagtaaaagtgatcaaatataaaaagattatcaTTTGTATAGTTCATCCATGC
S0015	GAGTAACTCGGCTGTCGGCTGTCGG
S0016	AATGGGAAGTATTCGGACGGGCGGAG

^a^Homology arm and PCR template annealing sequences given in lower and upper case, respectively

#### PCR-amplification of the *rps*L-*tet*A(C) and *trfA *recombineering cassettes

The *rps*L-*tet*A(C) counter-selection cassette (RT-cassette) [5] was generated by PCR using a gel-purified 2.7 kb *Nco*I fragment of pBAC-RT [13] as template (5 ng) and ODNs 6090 and 6091 (Table 1). These "recombineering" ODNs were PAGE-purified (IDT, Leuven, Belgium) and contain 50-nt 5' homology arms corresponding to sequences directly flanking the single *Nco*I and *Hin*dIII sites of pBADcre [11] (Fig. 1A), respectively, and designed to enable replacement of the *P*_BAD_-driven *cre *gene in EL350 [11] with *trfA*. The *trfA *replacement cassette was generated by PCR using pMW010 DNA (5 ng) as template and ODNs 6088 and 6089 (Table 1) that contained homology arms equivalent to those of 6090 and 6091 (Table 1) (Fig. 1B). Both PCR products were purified (Wizard SV columns, Promega) then quantified, by visualisation against a DNA mass ladder (1 kb ladder, NEB), prior to use.

#### Recombineering-mediated generation of MW005

Heat-shock-mediated induction of Red activities, preparation of electrocompetent *E. coli *cells and all subsequent recombineering steps were performed essentially as described [5]. Briefly, non-induced (control) or Red-induced electrocompetent EL350 cells were electroporated (Eppendorf 2510) with 500 ng of the PCR-generated RT-cassette and cells recovered (SOC [-Mg] medium, 32°C, 220 rpm, 2.5 h). Recovered cells were serially diluted (M9 salts), aliquots (50 μl) spread on either selective (Tc, 5 μg/ml) or non-selective (for cell viability determination) LB-agar plates and incubated (32°C, 48 h). Five discrete Tc-resistant colonies were re-streaked on Tc-selective LB-agar plates and incubated further (32°C, 48 h). Those colonies in which the RT-cassette had been inserted correctly into the host chromosome were identified by colony-PCR using the insertion site-flanking ODNs 6102 and 6103 (Table 1). The RT-cassette-containing EL350 strain was named MW004.

In the subsequent replacement recombineering step the PCR-generated *trfA *gene sequence (500 ng) was introduced into non- or Red-induced MW004 cells by electroporation and, subsequently, recovered cells were spread on either selective (Sm, 500 μg/ml) or non-selective LB-agar plates and incubated (32°C, 48 h). As above, a number of discrete Sm-resistant colonies were re-streaked to fresh Sm-selective plates and incubated further (32°C, 48 h). Those colonies in which the RT-cassette had been replaced with the *trfA *sequence were identified, initially by colony-PCRs, with ODNs 6102 and 6103 (Table 1) and subsequently by direct sequencing of the resulting PCR products. The new strain, MW005, has the *P*_BAD_-driven chromosomal *cre *sequence replaced with that encoding *trfA*.

#### Copy-number induction of pCC1FOS- and pCC1BAC-based genomic clones in MW005

Construct fUL#SB28 (see below), two additional *C. elegans *pCC1FOS-based genomic clones WRM0636aA04 and WRM067aC01 and *Lates calcarifer *pCC1BAC-based genomic clones A02_CBP0333, A10_CBP1191 and H12_CBP0642 [19] were electroporated into MW005 and EPI300 and recovered bacteria plated on selective (Chl, 12.5 μg/ml) LB-agar plates. Aliquots of overnight cultures (10 ml LB, Chl 12.5 μg/ml, 32°C, 220 rpm), each inoculated with a single colony, were mixed with fresh medium (LB, Chl 12.5 μg/ml) to give a final volume of 20 ml and OD_600 _of 0.4. Each diluted culture was divided equally into two 10 ml aliquots with 10 μl of CopyControl induction solution (Epicentre) being added to one aliquot before all were incubated for an additional 4 h (32°C, 220 rpm). Cell densities (OD_600_) were measured enabling DNA to be isolated, by standard alkali lysis and isopropanol precipitation, from the same number of bacterial cells (equivalent to 5 ml at an OD_600 _of 3.0) per culture. DNA was resuspended (50 μl TE) and aliquots (5 μl) incubated with either *BamH*I or *Nco*I. Non-diluted and 2-fold serial dilutions of the digested DNA from, respectively, non-copy-number-induced and copy-number-induced cells were subject to agarose gel (0.7% w/v) electrophoresis (Fig. 2).

### Construction of a fosmid-based *ceh-12::gfp *reporter gene fusion in MW005

#### Recombineering

The generation of the linear RT and *gfp *replacement cassettes differed slightly from that described above and previously [5] in that the counter-selection RT-cassette was flanked by 50 bp regions corresponding to the extreme 5' and 3' ends of the *gfp *coding sequence. This enabled the replacement *gfp *cassette to be provided as a simple restriction-fragment excised from a suitable *gfp*-containing plasmid [10]. Briefly, a target specific [5-*gfp*-RT-*gfp-*3']-cassette was amplified by PCR using, as a template, a plasmid containing a 5'-*gfp*-RT-*gfp-*3' sequence [10], and ODNs S0316 & S0317 (Table 1). These primers contained 50-nt 5' homology arms designed to insert the [5'-*gfp*-RT-*gfp*-3']-cassette immediately upstream of the translation termination codon of the *C*. *elegans ceh-12 *gene. The amplified [5'-*gfp*-RT-*gfp*-3']-cassette was electroporated into recombineering-competent MW005 *E*. *coli *that had been previously transformed with the pCC1FOS-based genomic clone, WRM0611dH08. The 33.5 kb insert of this clone contains the entire *ceh-12 *locus plus significant 3' (>25 kb) and 5' (>6 kb) flanking genomic DNA (Fig. 3A). Following recovery, cells were spread on Tc-selective LB-agar plates and restreaked. Fosmids containing a [5'-*gfp*-RT-*gfp*-3']-cassette inserted correctly at the target site were identified by colony-PCR using the insertion site-flanking ODNs S0015 and S0016 (Table 1). Replacement of the 5'-*gfp*-RT-*gfp*-3' sequence with *gfp *within electro- and recombineering-competent MW005 was carried out as described above except a restriction fragment, encoding the complete *gfp *coding sequence, minus the termination codon, was used [10] and cells were selected on LB-agar plates containing both Sm (500 μg/ml) and chloramphenicol (Cm) (12.5 μg/ml). A recombinant fosmid clone containing the correctly recombineered *ceh-12*::*gfp *fusion sequence was identified by colony-PCR, using PCR primers flanking the insertion site, and detailed restriction digestion (data not shown) and was called fUL#SB28 (Fig. 3C). Sequencing across the insertion site was deemed unnecessary as the replacement sequence was introduced into MW005 as a restriction fragment [10] rather than a PCR product as used previously [5].

#### *C. elegans *transformation and observation

Following copy-number induction, performed as described above, and DNA isolation transformation of *C*. *elegans *N2 worms with fUL#SB28 and plasmid pRF4 was performed by microinjection into adult syncytial gonads as described [5]. GFP expression in adult hermaphrodites from independently derived lines was observed by fluorescence microscopy as described (Fig. 3D) [5].

## Authors' contributions

MW constructed and HS helped validate MW005. SB constructed the translational GFP reporters *via *recombineering in MW005. IAH supervised SB. CTD supervised MW and HS and wrote the manuscript. All authors have read and approved the final manuscript.
